# A dataset of publications and patents on quantum error correction (1995–2025)

**DOI:** 10.1016/j.dib.2026.113149

**Published:** 2026-08-06

**Authors:** Saikat Barua, Humayra Shemushee

**Affiliations:** a Department of Electrical and Computer Engineering, North South University, Plot 15, Block B, Bashundhara R/A, Dhaka, 1229, Bangladesh; b SpontAlign, Dhaka, Bangladesh

**Keywords:** Scientometrics, Bibliometrics, Patent analysis, Fault-tolerant quantum computing, Research output, Text corpus, Open data, OpenAlex

## Abstract

This article presents a harmonized corpus of scholarly publications and patents concerning quantum error correction, the encoding techniques that protect quantum information against noise and that underpin the pursuit of fault-tolerant quantum computation. The corpus spans 1 January 1995 to 31 December 2025 and contains 16,274 records: 14,385 scholarly publications harvested from OpenAlex and enriched from Crossref, and 1889 United States patent publications retrieved from Google Patents Public Data. Publications were collected by matching twenty scope phrases against titles and abstracts, intersected with a quantum-computing topic to suppress unrelated matches; patents were collected under the Cooperative Patent Classification class for quantum error correction. Both record types were mapped onto a common eighteen-field schema, deduplicated within type, and assigned to one of three retrieval tiers according to the strength of the topical match. Each record carries subfield tags and flags marking machine-learning decoder records and adjacent-topic mentions. The precision of the core tier was assessed by manual annotation of a stratified sample, and inter-annotator agreement was measured. The corpus, its variable dictionary, provenance metadata, validation samples, and the complete construction notebook are openly available, supporting reuse in scientometrics, patent analysis, and text mining of the field.

Specifications table

**Table:** 

Subject	Computer Sciences
Specific subject area	Scientometrics and patent analysis of quantum error correction and fault-tolerant quantum computing.
Type of data	Table (CSV and JSONL format). Raw and filtered.
Data collection	Records were harvested programmatically from two open sources. Publications were retrieved from the OpenAlex REST API using twenty scope phrases matched against titles and abstracts, each query intersected with the OpenAlex quantum-computing topic (T10682); missing venue names were completed via the Crossref API. Patents were retrieved from Google Patents Public Data through Google BigQuery, covering United States patent publications under CPC class G06N10/70. Records were mapped to a common schema, deduplicated within record type, and assigned to one of three retrieval tiers. No proprietary databases were used.
Data source location	Data processing was performed at: Institution: North South University City/Region: Dhaka Country: Bangladesh The dataset is secondary data compiled from the following primary sources: • OpenAlex, OpenAlex API (openalex.org), CC0 — scholarly publication metadata • Crossref, Crossref REST API (crossref.org) — venue metadata • Google Patents Public Data, accessed via Google BigQuery (dataset patents-public-data.patents.publications) — United States patent publications
Data accessibility	Repository name: Mendeley Data Data identification number: 10.17632/cxrrmvy56p.2 Direct URL to data: https://doi.org/10.17632/cxrrmvy56p.2 Instructions for accessing these data: The dataset is publicly available under a CC BY 4.0 licence. The repository contains the harmonized corpus (qec_corpus.csv and qec_corpus.jsonl), the variable dictionary, provenance metadata, validation samples, the raw patent extract, and the construction notebook. No access controls apply.
Related research article	None.

## 1 Value of the Data

• These data provide a single, openly licensed corpus that combines scholarly publications and patents for quantum error correction, the field of techniques that protect quantum information against noise and underpins the pursuit of fault-tolerant quantum computing. Publications and patents are mapped onto one common schema, so academic and industrial output can be examined together without further reconciliation.
• The data can be reused by scientometricians, science-policy analysts, research managers, and historians of science and technology to characterize output growth, subfield composition, publication venues, contributing institutions, patent assignees, and the relationship between academic research and industrial invention in a rapidly expanding area of quantum computing.
• Each record carries a retrieval tier recording the strength of its topical match, and the precision of every tier has been measured by manual audit: 86.0% in the core tier (Wilson 95% confidence interval 77.9–91.5%), 37.0% in the extended tier (28.2–46.8%), and 2.0% in the weak patent tier (0.6–7.0%). Two annotators labeled each audited sample independently, and the labeled samples are supplied so that users can inspect the boundary judgements and apply their own criteria.
• A labeled subset identifies records concerned with machine-learning and neural decoders, so researchers studying the adoption of machine-learning methods within quantum error correction can locate the relevant records without screening the full corpus. Titles and abstracts are included, supporting reuse as a domain-filtered text collection for natural language processing and information-retrieval experiments.
• The corpus records that 1247 of 1889 patents (66.0%) carrying the quantum-error-correction patent class CPC G06N10/70 contain no quantum-error-correction term in their title or abstract; these records are retained and labeled rather than removed, so that researchers assembling patent datasets in this field can see the composition of the class and choose their own screening rule.

## 2 Background

Quantum error correction encodes quantum information redundantly across physical qubits so that errors can be detected and corrected without disturbing the encoded state, and it is a prerequisite for fault-tolerant quantum computation [[1], [2]–3]. Foundational schemes established that quantum information could be protected using quantum analogues of classical error-correcting codes [1,2], and subsequent work developed stabilizer [4] and topological codes, fault-tolerant protocols, and surface-code architectures suited to physical hardware [[5], [6]–7], with recent hardware demonstrations operating below the surface-code threshold [8]. Research activity now spans decoders, bosonic and quantum low-density parity-check codes [9], and magic-state distillation [10], and it is accompanied by a growing body of patents filed by academic and industrial organizations, classified under a dedicated patent scheme.

Bibliographic records of this activity are distributed across separate systems: scholarly publications are indexed by open services such as OpenAlex [11] and Crossref [12], while patents are held in patent-office collections such as Google Patents Public Data. These systems use different identifiers, fields, and classification schemes, and much prior scientometric work in adjacent areas [13] has relied on proprietary databases whose underlying records cannot be redistributed. This dataset was compiled to bring the publication and patent record of quantum error correction together in a single, openly licensed, harmonized corpus with a documented and reproducible construction pipeline, covering the period from 1995 to 2025. The dataset does not support a related research article.

## 3 Data Description

This article describes the harmonized corpus of quantum error correction publications and patents, openly available at Mendeley Data [14] (see Specifications Table). The repository contains the corpus in two formats, its documentation, the validation materials, the raw patent extract, and the construction notebook. An overview of every file is given in Table 1; the variables within the corpus are defined in Table 2.

**Table 1:** Content of the repository: files, formats, and their contents.

File	Format	Content
qec_corpus.csv	CSV	The harmonized corpus: 16,274 records × 18 fields (see Table 2).
qec_corpus.jsonl	JSONL	The same corpus, one JSON object per record.
data_dictionary.json	JSON	Machine-readable dictionary of the 18 variables, with completeness reported separately for publications and patents.(as in Table 2)
dataset_metadata.json	JSON	Dataset title, snapshot date, coverage window, licence, tier definitions, per-source provenance, and validation summary.
README.md	Markdown	Description of the dataset, the tier scheme, and the audited precision of each tier.
precision_audit_v2_labeled.csv	CSV	The 100-record stratified core-tier validation sample with manual in-scope labels.
precision_audit_consensus.csv	CSV	The same core-tier sample with two annotators' labels and the reconciled consensus label.
google_patents_qec_raw.csv	CSV	Unprocessed rows returned by the BigQuery patent query, before harmonization.
T2_field_dictionary.csv	CSV	The same variable dictionary in tabular form (as in Table 2).
scope_definition.json	JSON	The twenty scope phrases with their subfield mappings, tier rules, and the CPC class.
provenance.json	JSON	Execution environment, retrieval parameters and dates, scope, validation summary, and file checksums.
requirements_freeze.txt	Text	Exact package versions of the environment in which the corpus was produced.
checksums.sha256	Text	SHA256 checksum for every file in the repository.
precision_audit_tier2.csv	CSV	The 100-record extended-tier validation sample with both annotators' labels and the consensus label.
precision_audit_tier3.csv	CSV	The 100-record weak-tier validation sample with both annotators' labels and the consensus label.
precision_audit_disagreements_resolved.csv	CSV	CSV Every annotator disagreement across the three tiers, with the written reason for its resolution.
data-in-brief-qec-main.ipynb	Jupyter notebook	The pipeline that produces the corpus end to end: retrieval, harmonization, tiering, and validation.

**Table 2:** Variables in the corpus, with descriptions and per-field completeness reported separately for publications (*n* = 14,385) and patents (*n* = 1889). Fields marked "Publications only" are structurally absent for patents and are reported as not applicable rather than as missing values.

Variable	Description	Applies to	Complete, publications (%)	Complete, patents (%)	Complete, all records (%)
record_id	OpenAlex work ID or patent number	Both	100	100	100
Source	openalex or google_patents_public_data	Both	100	100	100
type	publication or patent	Both	100	100	100
tier	Retrieval tier: 1 core, 2 extended, 3 weak	Both	100	100	100
doi	Digital Object Identifier	Publications only	91.2	n/a	n/a
title	Record title	Both	100	100	100
abstract	Abstract text	Both	87.2	100	88.7
year	Publication or patent year	Both	100	100	100
work_type	Article, preprint, patent publication, etc.	Both	100	100	100
venue	Journal or issuing office	Both	97.0	100	97.3
cited_by	Citation count	Publications only	100	n/a	n/a
authors	Authors or assignees, with ORCID and affiliation	Both	99.4	100	99.5
n_authors	Number of authors or inventors	Both	100	100	100
references	Cited work identifiers	Publications only	65.7	n/a	n/a
subfields	Pipe-separated subfield tags	Both	100	100	100
matched_terms	Retrieval phrases or CPC codes matched	Both	100	100	100
exclude_flag	Mentions an adjacent topic (advisory)	Both	100	100	100
ml_decoder	Concerns machine-learning or neural decoders	Both	100	100	100

The corpus (qec_corpus.csv and the equivalent qec_corpus.jsonl) contains 16,274 records, each a scholarly publication (14,385 records) or a United States patent publication (1889 records) concerning quantum error correction, covering 1 January 1995 to 31 December 2025. Each record carries the 18 fields defined in Table 2, together with the completeness of each field. Field completeness is reported separately for publications and patents in Table 2, because several fields apply to only one record type. The DOI, citation count, and reference-list fields are populated for scholarly publications but are structurally absent for patents, which carry no such attributes in the source data; these are reported as not applicable for patents rather than as missing values. Within publications, the DOI is present for 91.2% of records, absent chiefly for preprints and conference items without a registered DOI, and reference lists are present for 65.7%, absent where OpenAlex holds no reference data for the record. Fields populated for both record types are occasionally empty where the source record itself lacked the value: abstracts are present for 87.2% of publications and all patents, and a venue or issuing office for 97.0% of publications and all patents. Identifier, title, year, and record type are complete throughout.

**Fig. 1:**
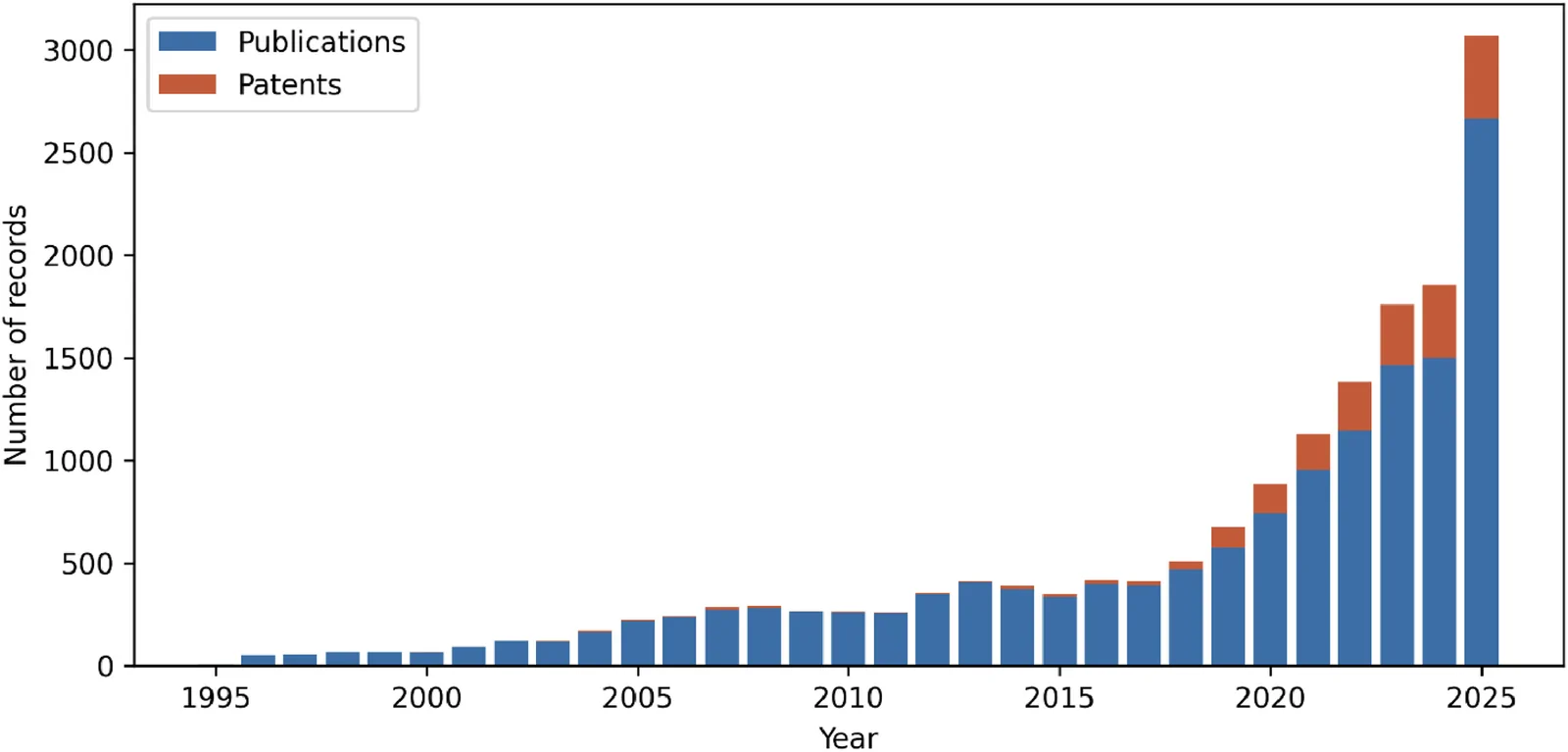
Annual number of quantum error correction publications and patents in the corpus, 1995–2025.

**Fig. 2:**
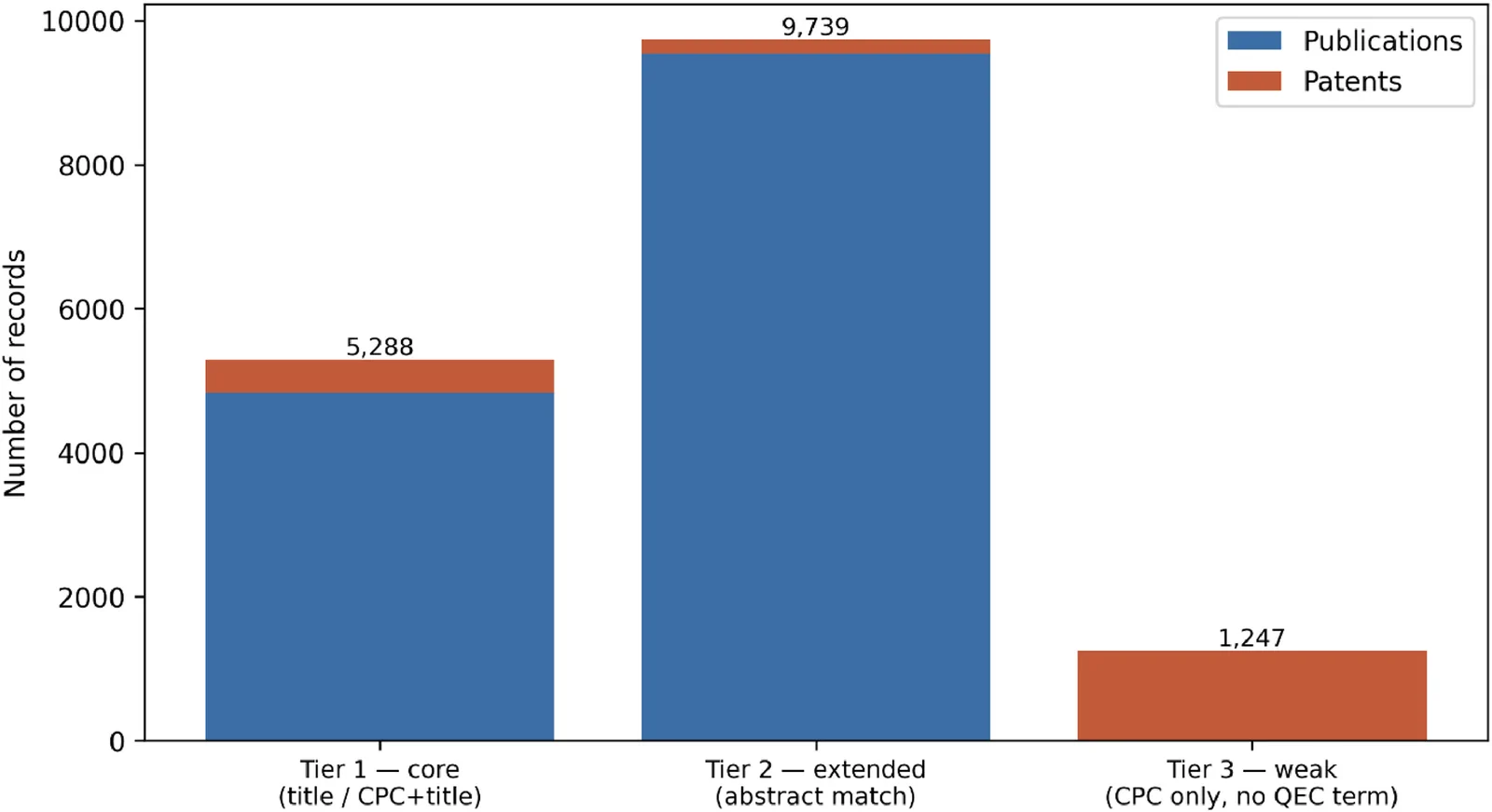
Number of records by retrieval tier and record type.

Every record is assigned to one of three retrieval tiers, given in the tier field: a core tier (5288 records), an extended tier (9739 records), and a weak tier of patents only (1247 records). The number of records per year is shown in Fig. 1, and the distribution across tiers and record types in Fig. 2.

**Fig. 3:**
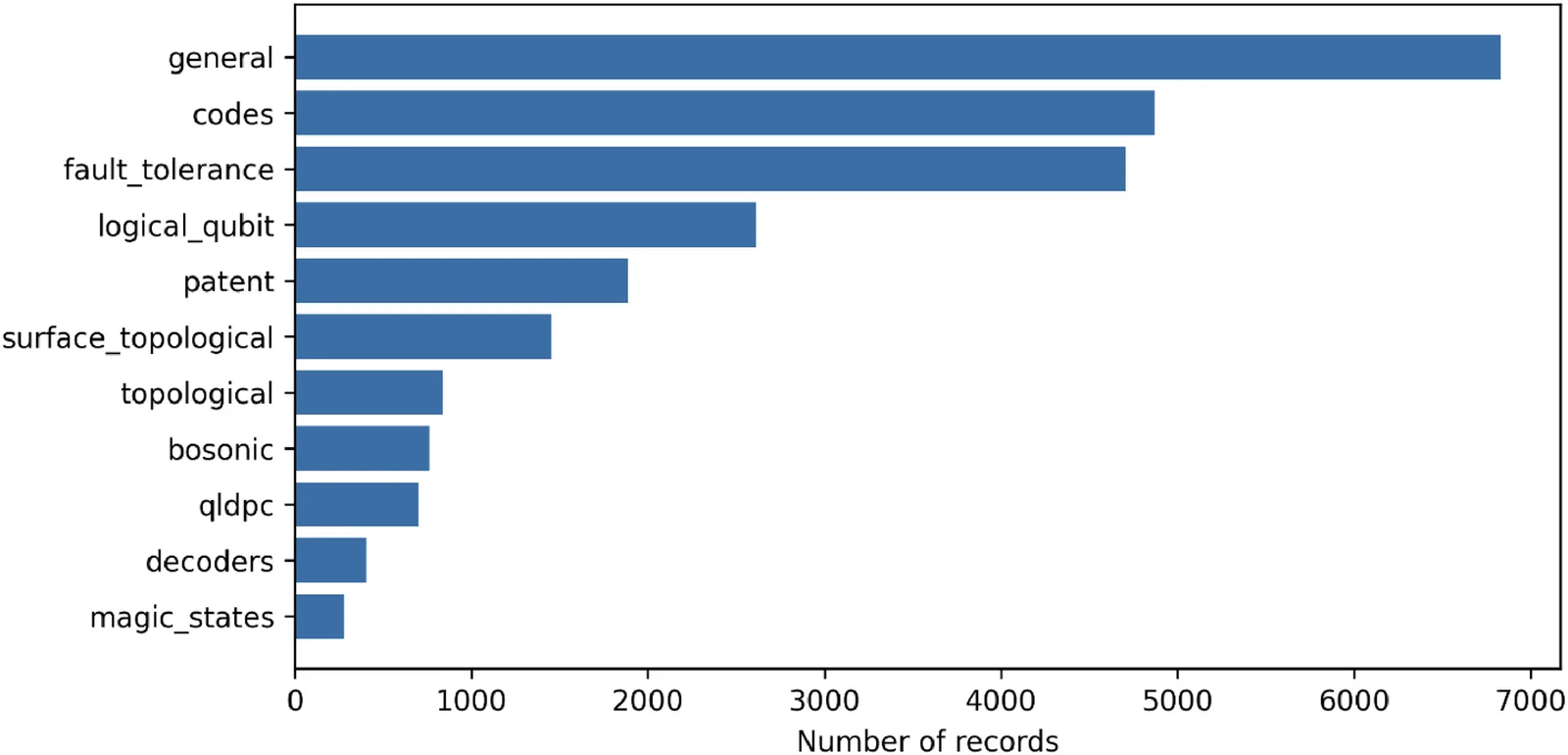
Distribution of records across quantum error correction subfield tags. Records may carry more than one tag, so the counts sum to more than the number of records.

Each record carries one or more subfield tags in the subfields field (for example codes, fault_tolerance, surface_topological, bosonic, qldpc, magic_states, decoders, logical_qubit, patent, general), indicating which aspects of quantum error correction it concerns. Because a record may carry several tags, the tag counts sum to more than the number of records; their distribution is shown in Fig. 3. A boolean ml_decoder field marks the subset of records concerning machine-learning and neural decoders, and a boolean exclude_flag field marks records whose text mentions an adjacent topic (such as error mitigation or quantum key distribution). Each scope phrase maps to one subfield tag, and a record receives the union of the tags of every phrase it matched; tags are therefore overlapping rather than mutually exclusive, and every record carries at least one tag. The exclude_flag is independent of the subfield tags: it marks records whose text mentions an adjacent topic, and a record may carry both a subfield tag and the flag, since reviews of quantum error correction frequently mention error mitigation or quantum key distribution in passing.

All 1889 patents carry the Cooperative Patent Classification class G06N10/70. Their distribution across the three tiers, according to whether a quantum-error-correction term appears in the title, in the abstract only, or nowhere in the title or abstract, is given in Table 3.

**Table 3:** Screening outcome for the 1889 patents carrying CPC class G06N10/70, by whether a quantum-error-correction term appears in the title, in the abstract only, or nowhere.

Screen	Tier	Patents	Percent
CPC G06N10/70; QEC term in title	1	449	23.8
CPC G06N10/70; QEC term in abstract only	2	193	10.2
CPC G06N10/70 only; no QEC term anywhere	3	1247	66.0

The validation materials are provided as five files. precision_audit_v2_labeled.csv and precision_audit_consensus.csv contain the core-tier sample with the manual in-scope judgement and with both annotators' independent labels. precision_audit_tier2.csv and precision_audit_tier3.csv contain the corresponding samples for the extended and weak tiers. precision_audit_disagreements_resolved.csv records every disagreement together with the written reason for its resolution. Precision was estimated for every retrieval tier, not for the core tier alone; the estimates and the inter-annotator agreement for all three tiers are given in Table 4.

**Table 4:** Precision and inter-annotator agreement for each retrieval tier, from stratified random samples of 100 records per tier with Wilson 95% confidence intervals.

Tier	n	In scope	Precision	95% CI low	95% CI high	Percent agreement	Cohen's κ	Disagreements
1 — core	100	86	0.860	0.779	0.915	97.0	0.863	3
2 — extended	100	37	0.370	0.282	0.468	82.0	0.649	18
3 — weak	100	2	0.020	0.006	0.070	82.0	0.151	18

Precision declines across the three tiers, and the confidence intervals do not overlap. The inter-annotator agreement for the weak tier is deflated by the highly skewed marginal label distribution, since only two of the hundred sampled records were judged in scope; percent agreement is reported alongside Cohen's κ for this reason. All disagreements in the extended and weak tiers concerned records at the boundary between correction and suppression or mitigation.

## 4 Experimental Design, Materials and Methods

**Fig. 4:**
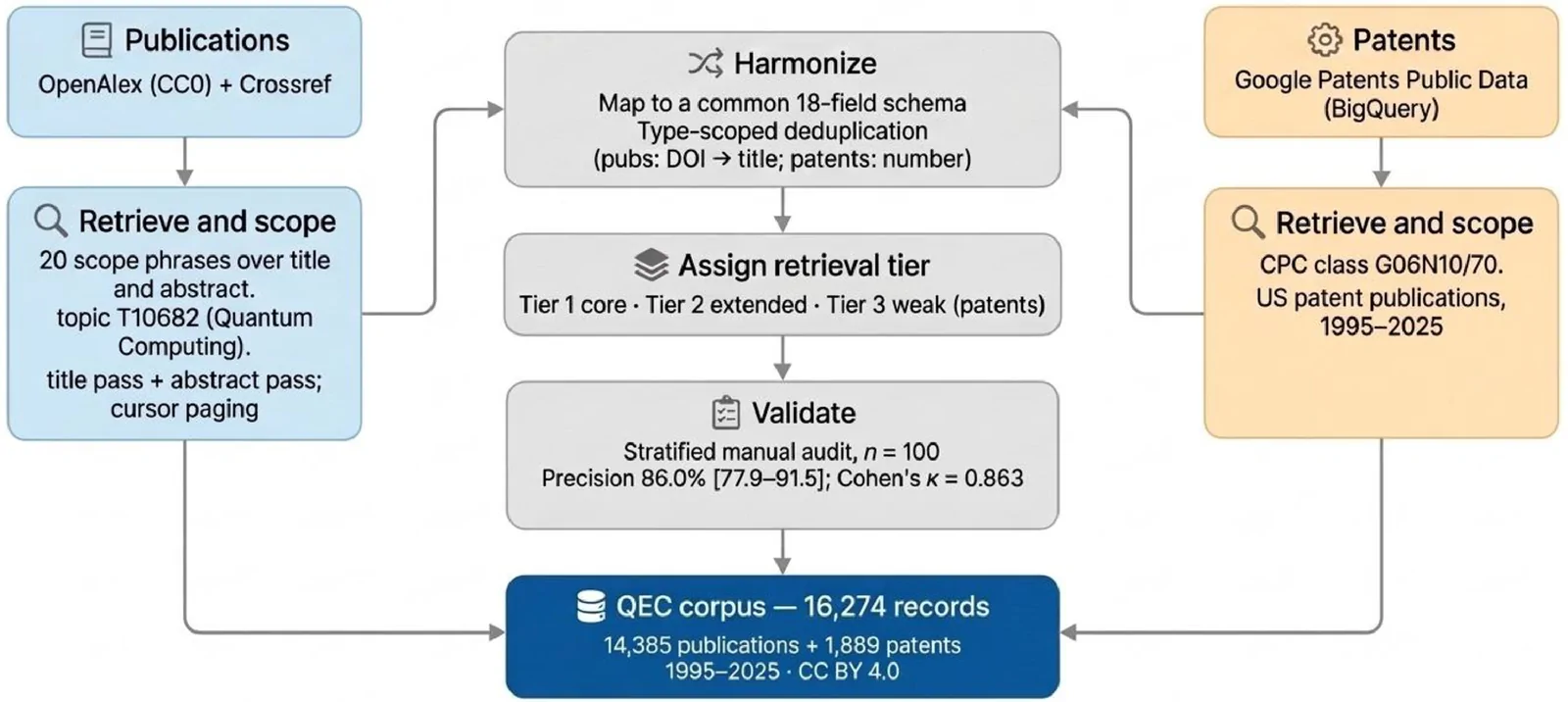
Workflow for constructing the corpus. Publications (OpenAlex, enriched from Crossref) and patents (Google Patents Public Data via BigQuery) were harmonized to a common schema, deduplicated within record type, assigned to retrieval tiers, and validated by a manual precision audit.

The corpus was assembled by a deterministic, four-stage pipeline: publication retrieval from OpenAlex, venue enrichment from Crossref, patent retrieval from Google Patents Public Data, and harmonization with tiering and validation. An overview of the pipeline is shown in Fig. 4. The complete pipeline is provided as the Jupyter notebook data-in-brief-qec-main.ipynb in the repository, and was executed on the Kaggle Notebooks platform (Python 3.12.13) with internet access enabled. The principal package versions were pandas 2.3.3, numpy 2.0.2, requests 2.32.4, scikit-learn 1.6.1, matplotlib 3.10.0, and google-cloud-bigquery 3.41.0; the complete environment is recorded in requirements_freeze.txt, and the retrieval parameters and SHA256 checksums of every deposited file in provenance.json and checksums.sha256. All records were harvested over a fixed snapshot window of 1 January 1995 to 31 December 2025; the snapshot date is recorded in dataset_metadata.json. No proprietary bibliographic databases were used.

### 4.1 Scope definition

The topical boundary of quantum error correction was defined by twenty scope phrases, each mapped to a subfield tag. Eleven were treated as anchor phrases specific to the field (for example quantum error correction, stabilizer code, magic state distillation, quantum LDPC, lattice surgery, GKP code, quantum syndrome decoding); nine were treated as ambiguous phrases that also occur in adjacent fields (for example surface code, logical qubit, toric code, color code, cat qubit, concatenated code, subsystem code). Quantum key distribution, error mitigation, and classical channel coding were defined as adjacent and out of scope. The full phrase-to-tag mapping is contained in the notebook.

### 4.2 Publication retrieval

Publications were retrieved from OpenAlex [11], an open, fully accessible bibliographic index whose coverage differs from that of proprietary databases such as Web of Science and Scopus [15], through its REST API. Each scope phrase was queried over record titles and abstracts, and every query was intersected with the OpenAlex topic Quantum Computing Algorithms and Architecture (topic identifier T10682) so that token-level matches from unrelated disciplines were excluded. Two retrieval passes were run for every phrase: a title pass, in which the phrase was required to appear in the record title, and a title-and-abstract pass, in which the phrase was required to appear in the title or abstract. Results were paginated with cursor paging and union across all phrases; duplicate works were merged on the OpenAlex work identifier, and a record retrieved in more than one pass was retained once. For each record, the identifier, DOI, title, publication year, work type, host venue, authorships (author names, ORCID identifiers, and institutional affiliations), citation count, and referenced works were retained, and the abstract was reconstructed from the OpenAlex inverted index. Requests were transmitted through the OpenAlex polite pool using an authenticated key, at 200 records per page. Rate-limited responses were retried with exponential back-off honouring the Retry-After header, and the harvest state was checkpointed atomically every five pages so that an interrupted run could resume without re-querying.

### 4.3 Venue enrichment

Records carrying a DOI but no host-venue name after the OpenAlex pass were enriched against the Crossref REST API [12]. For each such record, the container title returned by Crossref was used to complete the venue field. Records without a DOI, and records whose venue was already present, were not queried.

### 4.4 Patent retrieval

Patents were retrieved from the Google Patents Public Data collection through Google BigQuery, querying the table patents-public-data.patents.publications. United States patent publications with a publication date within the snapshot window and carrying the Cooperative Patent Classification class G06N10/70, "Quantum error correction, detection or prevention", were selected. For each patent the publication number, family identifier, publication and filing dates, localized title and abstract, Cooperative Patent Classification codes, harmonized assignee names, and harmonized inventor names were retained. The unfiltered query result is provided as google_patents_qec_raw.csv. Records represent patent publications; no patent-family deduplication was applied.

### 4.5 Harmonization and tiering

Publications and patents were mapped onto a single record schema. Deduplication was performed within record type: publications were deduplicated by DOI and then by normalized title, and patents by publication number; publications and patents were never deduplicated against one another. Each record was then assigned to one of three retrieval tiers. For publications, the core tier comprises records whose title matched a scope phrase, and the extended tier comprises records matched only in the abstract. For patents, the core tier comprises records with a quantum-error-correction term in the title, the extended tier comprises records with such a term in the abstract only, and the weak tier comprises records carrying the class G06N10/70 without any such term in the title or abstract. Each record was tagged with its subfield or subfields, a Boolean flag marking mention of an adjacent topic, and a Boolean flag marking records concerned with machine-learning and neural decoders. The harmonized corpus was written to qec_corpus.csv and qec_corpus.jsonl.

### 4.6 Validation

Precision was estimated for each of the three retrieval tiers from a stratified random sample of 100 records per tier, drawn under a fixed seed of 42. The core-tier sample comprises 75 publications and 25 patents, a ratio chosen to over-represent patents relative to their share of the tier so that the patent layer could be estimated separately with a usable interval. The extended-tier sample was drawn in proportion to the composition of that tier, giving 98 publications and 2 patents, and the weak-tier sample comprises 100 patents, that tier containing no publications.

Two annotators screened every sampled record independently. A record was judged in scope only if it is primarily concerned with encoding quantum information into a code, syndrome extraction, and correction. Error mitigation, error suppression, dynamical decoupling, composite and optimal control pulses, leakage removal, quantum key distribution, and algorithms designed to run on fault-tolerant machines were judged out of scope.

Precision was computed as the proportion of sampled records judged in scope. Because the sample is small, confidence intervals were computed using the Wilson score interval rather than the normal approximation. For n sampled records of which k are judged in scope, the observed precision is p̂ = k/n, and the confidence interval at level 1 − α is given by Eq. (1):

(1) $$\hat{p}\pm =\left(\hat{p}+z^{2}/\left(2n\right)\pm z\surd \left(\hat{p}\left(1-\hat{p}\right)/n+z^{2}/\left(4n^{2}\right)\right)\right)/\left(1+z^{2}/n\right)$$

where z is the standard-normal quantile for the chosen confidence level (*z* = 1.96 for 95% intervals) [16].

Inter-annotator agreement between the two annotators was quantified with Cohen's κ, defined in Eq. (2):

(2) $$\kappa =\left(p_{o}-p_{e}\right)/\left(1-p_{e}\right)$$

where p_o_ is the observed proportion of records on which the two annotators agree and p_e_ is the proportion of agreement expected by chance, computed from the annotators' marginal label frequencies [17]. A value of κ = 1 indicates perfect agreement, and κ = 0 indicates agreement no greater than chance.

Disagreements were resolved jointly by discussion against the scope rule rather than by deferring to either annotator, and each resolution is recorded with its written reason in precision_audit_disagreements_resolved.csv. A large language model produced provisional screening labels for the core-tier sample only. Every record in every tier was subsequently labeled independently by both annotators, and the model's labels do not enter any reported figure.

## Limitations

The dataset has several limitations relating to coverage and construction. The patent layer is restricted to United States patent publications; patents filed only in other jurisdictions are not represented, so the corpus understates non-US invention activity. Patent records are counted as individual publications rather than as unique inventions, because no patent-family deduplication was applied, so a single invention with multiple publications appears more than once. Coverage of the publication layer depends on OpenAlex and Crossref, and inherits any indexing gaps, absent abstracts, or incomplete affiliation and reference data present in those sources; per-field completeness is reported in Table 2 and reflects both fields that apply only to publications and records for which the source lacked a value. Because OpenAlex indexes preprints alongside published articles, the share of preprints is higher in recent years, which affects the yearly record counts. The topical boundary is defined by English-language scope phrases and by a single OpenAlex topic, so relevant records described only in other languages, or not assigned that topic, may be omitted. Recall was not measured. No exhaustive reference set of quantum error correction records exists for this window, so the tiers describe the strength of the topical match at retrieval rather than a measured trade-off between precision and recall. The audited precision of the extended tier is 37.0% and that of the weak tier 2.0%, so the majority of records in those tiers are not primarily concerned with quantum error correction; the core tier is recommended for analyses requiring topical precision, and the audited samples and labels are provided so that users can assess and adjust the boundary for their own purposes.

## Ethics Statement

The authors have read and follow the ethical requirements for publication in Data in Brief and confirm that the current work does not involve human subjects, animal experiments, or any data collected from social media platforms.

## CRediT Author Statement

Saikat Barua: Conceptualization, Methodology, Software, Data curation, Validation, Writing – original draft. Humayra Shemushee: Conceptualization, Validation, Writing – review & editing.
